# Plantaricin NC8 αβ prevents *Staphylococcus aureus*-mediated cytotoxicity and inflammatory responses of human keratinocytes

**DOI:** 10.1038/s41598-021-91682-6

**Published:** 2021-06-15

**Authors:** Amani Musa, Emanuel Wiman, Robert Selegård, Daniel Aili, Torbjörn Bengtsson, Hazem Khalaf

**Affiliations:** 1 School of Medical Sciences, Örebro University, Campus USÖ, Södra Grev Rosengatan 42A, 703 62 Örebro, Sweden; 2 Laboratory of Molecular Materials, Division of Biophysics and Bioengineering, Department of Physics, Chemistry and Biology (IFM), Linköping University, Linköping, Sweden

**Keywords:** Biochemistry, Cell biology, Microbiology

## Abstract

Multidrug resistance bacteria constitue an increasing global health problem and the development of novel therapeutic strategies to face this challenge is urgent. Antimicrobial peptides have been proven as potent agents against pathogenic bacteria shown by promising in vitro results. The aim of this study was to characterize the antimicrobial effects of PLNC8 αβ on cell signaling pathways and inflammatory responses of human keratinocytes infected with *S. aureus*. PLNC8 αβ did not affect the viability of human keratinocytes but upregulated several cytokines (IL-1β, IL-6, CXCL8), MMPs (MMP1, MMP2, MMP9, MMP10) and growth factors (VEGF and PDGF-AA), which are essential in cell regeneration. *S. aureus* induced the expression of several inflammatory mediators at the gene and protein level and PLNC8 αβ was able to significantly suppress these effects. Intracellular signaling events involved primarily c-Jun via JNK, c-Fos and NFκB, suggesting their essential role in the initiation of inflammatory responses in human keratinocytes. PLNC8 αβ was shown to modulate early keratinocyte responses, without affecting their viability. The peptides have high selectivity towards *S. aureus* and were efficient at eliminating the bacteria and counteracting their inflammatory and cytotoxic effects, alone and in combination with low concentrations of gentamicin. We propose that PLNC8 αβ may be developed to combat infections caused by *Staphylococcus* spp.

## Introduction

*Staphylococcus aureus (S. aureus)* is considered to be one of the most common skin-colonizing bacteria as well as a normal commensal found in the nasopharynx^1^. It is described to be the leading cause of bacterial infections in the skin and soft tissues, especially surgical site infections^2,3^. Virulence factors of *S. aureus* are e.g. the surface proteins that facilitate host-cell adhesion and act to evade host immune defences. Moreover, *S. aureus* has the ability to produce several toxins and proteases that make the infections difficult to treat^1^.

The skin in healthy humans provides a physical as well as an immunological barrier and constitutes an important protection against bacterial invasion^1^, including production of antimicrobial peptides in response to infections. Keratinocytes are the major cell type in the skin and are responsible for the initiation and orchestration of early cutaneous immune responses^4,5^. These cells express different pattern recognition receptors (PRRs) that sense pathogen-associated molecular patterns (PAMPs) of invading pathogens. Such receptors include toll like receptors (TLRs) and nucleotide-binding oligomerization domain proteins (NODs). Moreover, activation of protease-activated receptor 2 (PAR2) plays a crucial role in inflammatory processes of the skin^6^. Activation of these receptors induces signalling pathways, such as nuclear factor κB (NFκB) and mitogen-activated protein kinase (MAPK), resulting in the transcription of proinflammatory genes, for instance those encoding TNF, IL-6 and CXCL8^5^. These cytokines are found to influence immune responses by affecting the migration and proliferation of inflammatory cells. Furthermore, they have an impact on the proliferation and differentiation of keratinocytes, as well as their cytokine secretion^7^.

When the skin or mucous membrane is breached, the opportunistic *S. aureus* can enter the underlying tissue, consequently causing infections in healthy immunocompetent individuals as well as immunocompromised patients^1^. *S. aureus* can cause superficial skin infections such as impetigo, folliculitis, cellulitis, infected ulcers and wounds, but also invasive life-threatening infections including abscesses, pneumonia, osteomyelitis, meningitis, endocarditis and sepsis^5^.

The first line of treatment of bacterial infections is antibiotics. However, antibiotic treatment has been complicated by the development of multi-drug resistant bacterial strains. Bacterial resistance to antibiotics is a rapidly growing problem worldwide^8^. *S. aureus* has developed resistance to most common antibiotics due to an extensive use in both human and animal healthcare worldwide, with the consequent appearance of the Methicillin-resistant *Staphylococcus aureus* (MRSA) strain. MRSA are susceptible to only a few types of antibiotics^9^ currently in use and infections with MRSA are thus difficult to treat and expensive to the health care system^2^. The rate by which new families of antibiotics are discovered and made available for clinical use has decreased significantly, and there is an urgent need for novel antibiotics and alternative antimicrobial strategies to prevent and treat bacterial infections^10^.

Bacteriocins are antimicrobial peptides secreted by different types of bacteria and have in recent years emerged as interesting alternatives to conventional antibiotics. Bacteriocins are either synthesized ribosomally or non-ribosomally, using so called non-ribosomal protein syntheses, and some are extensively modified at the post-translation level^11,12^. Bacteriocins have been categorized into two major classes based on their physicochemical properties. Class I, known as the lantibiotic family contains modified peptides, while class II (non-lantibiotic family) includes non-modified peptides^13^. The lantibiotic peptides are small peptides (< 5 kDa) and have a different mode of action due to their sequence and structures. The linear lantibiotic peptides have a membrane-disrupting activity while globular peptides have cellular enzymatic action^14^. Class II is further divided into subgroups due to diversity of sequence and mode of action of the peptides^13^. The two-peptide Plantaricin NC8 αβ (PLNC8 αβ) is a bacteriocin which has been characterized as a part of the Class IIb subfamily, and is produced by *Lactobacillus plantarum* upon coculture with other gram positive bacteria^15^. The mechanism of action of PLNC8 αβ seems to depend on the complementary actions of the two peptides. Both peptides are amphipathic and have a net positive charge allowing the peptides to interact with negatively charged phospholipids in the cell membrane of target bacteria. The peptides are believed to aggregate and fold into secondary structures in the cell membrane, ultimately causing pore formation^16^. Although a heterogenous group of molecules, most bacteriocins show rapid antimicrobial effect even at micromolar concentrations, and differ from established antibiotics in their mechanism of action, which in combination with their low toxicity towards their host makes them interesting candidates for pharmacological use^17^. Since it is considered to be more difficult for bacteria to develop resistance against a mechanism involving membrane disruption, plantaricins (Pln) have gained more interest to be developed as alternatives/adjuvants to antibiotic therapy against bacterial infections.

We have previously shown that PLNC8 αβ effectively inhibits the gram-negative periodontal pathogen *Porphyromonas gingivalis*^18^. Additionally, we found that plantaricins are effective in killing the gram positive bacteria *S. aureus* and *S. epidermidis* and have additive or synergistic effects when combined with conventional antibiotics^19^. In order to further elucidate the antibacterial effects of PLNC8 αβ and lay a foundation for a possible future clinical application, the aim of this study was to characterize the effects of PLNC8 αβ on cell signalling and inflammatory responses of human keratinocytes infected with *S. aureus*.

## Methods

### Bacterial culture conditions

Methicillin-sensitive *S. aureus* ATCC 29,213 (Manassas, VA, USA) and methicillin-resistant *S. aureus* CCUG 35,601 (Culture Collection, University of Gothenburg, Sweden) were stored at − 80 °C. GFP-expressing *S. aureus* were a kind gift from Maria Lerm, Department of Biomedical and Clinical Sceinces, Linköping University. An aliquot from a frozen stock of each *S. aureus* strain was streaked onto a Luria Bertani (LB) agar plate and incubated at 37 °C overnight. Single bacterial colonies were picked and inoculated into 5 ml LB broth and cultivated overnight on a shaker (300 rpm) at 37 °C. Using viable count by culturing on agar plates, the bacterial concentration was adjusted to correlate with approximately 5 × 10^8^ CFU/ml. The bacteria were washed by centrifugation at 10,000 g for 5 min and bacterial pellets were resuspended in Dulbecco’s Phosphate Buffered Saline (DPBS, Gibco Ltd, UK).

### Determination of antimicrobial activity

The peptides (PLNC8 α [H_2_N-DLTTKLWSSWGYYLGKKARWNLKHPYVQF-COOH], PLNC8 β [H_2_N-SVPTSVYTLGIKILWSAYKHRKTIEKSFNKGFYH-COOH]) were purchased from GL Biochem (Shanghai) Ltd. Minimal inhibitory concentration (MIC) and minimal bactericidal concentration (MBC) of PLNC8 αβ were determined using the broth microdilution method. A two-fold serial dilution of PLNC8 αβ ranging from 50 to 0.78 µM was prepared in a 96-well microtiter plate followed by addition of MSSA and MRSA (5 × 10^5^ CFU/mL, diluted in LB broth). The antimicrobial activity of PLNC8 αβ is dependent on the complementary action of the two peptides at equimolar concentrations, a final concentration of 50 µM is achieved by mixing 50 µM PLNC8 α and 50 µM PLNC8 β. MIC was determined at 24 h, by visual inspection combined with spectroscopical analysis at 620 nm, as the lowest concentration at which bacterial growth is completely inhibited. MBC was determined by culturing all the wells at which bacterial growth was inhibited on agar plates and determining bacterial viability after 24 h of incubation at 37 °C. The lowest concentration at which no bacterial growth was observed was considered as the MBC.

The setup of the time-kill assay was identical to the microdilution method. Samples were collected from each well (10 µl) at the indicated time-points and serially diluted, and 10 µl of each sample was cultured in triplicates on agar plates for 24 h at 37 °C to determine the number of viable bacterial colonies.

The antimicrobial activity of PLNC8 αβ was further determined microscopically by using the fluorescent nucleic acid stain SYTOX™ Green to visualise the presence of bacterial lysis. Briefly, bacteria were washed and resuspended in DPBS (5 × 10^5^ CFU/well), followed by exposure to a two-fold serial dilution of PLNC8 αβ, ranging from 40 to 0 µM, in 96-well microtiter plates for 5 min. Images were captured with Olympus BX41 at 40 × magnification and the fluorescence intensity was analysed using the Software ImageJ (National Institutes of Health, USA).

Checkerboard assays were performed for all the bacterial strains used in the experiment, to evaluate the additive or synergistic relationship of PLNC8 αβ and Gentamicin.

### Cell culture conditions and experimental design

Human keratinocytes (HaCaT, CLS Cell Lines Services, 300,493) were cultured in T75-flasks using Dulbecco’s Modified Eagle Medium (DMEM), supplemented with 10% heat inactivated fetal bovine serum (FBS) (Gibco, Life technologies ™) and maintained at 37 °C, 5% CO_2_. The cells were used at passages 12–20. The optimal cell count for the experiments was determined to be 2 × 10^5^ cells/well. Keratinocytes were seeded in 24 well plates using DMEM supplemented with 10% FBS and incubated overnight at 37 °C, 5% CO_2_ to a nearly confluent monolayer.

The cells were stimulated with different concentrations of PLNC8 αβ (6.25, 12.5 and 25 μM) and/or infected with increasing multiplicity of infection of *S. aureus* (MOI 0.1, 1 and 10) for 24 h to determine the dose response effects on human keratinocytes. Supernatants were collected and stored at − 20 °C to determine the levels of IL-6 and CXCL8. Furthermore, early and long terms effects of PLNC8 αβ on *S. aureus*-mediated cytotoxicity and inflammatory responses of the keratinocytes were investigated. At the start of the experiment, the culture media were replaced with fresh media without FBS, and the cells were incubated for 2 h prior to infection. The cells were either stimulated with PLNC8 αβ (6.25, 12.5 and 25 μM) or infected with *S. aureus* (MOI 1) for 1 h and then exposed to different concentrations of PLNC8 αβ (1.5, 3.1, 6.25, 12.5 and 25 μM) and incubated for 6 or 24 h, after which cell morphology was determined by microscopic evaluation and images were captured. The supernatants were collected and stored in -20 °C for further analysis and the cells were used for RNA extraction.

### Analysis of intracellular signaling

The NF-κB and MAPK pathways are important regulators of inflammatory responses. The role of these pathways in initiating inflammation of human keratinocytes after infection with *S. aureus*, in the presence or absence of PLNC8 αβ, was investigated by using specific inhibitors: NF-κB inhibitor (Bay 11–7085, R&D Systems), InSolution JNK Inhibitor II and ERK Inhibitor II (FR180204, Calbiochem). The inhibitors were added, at a final concentration of 10 µM, 2 h prior to infection with *S. aureus* and addition of PLNC8 αβ.

### LDH cytotoxicity assay

Cytotoxicity of *S. aureus* and PLNC8 αβ on HaCaT cells was determined by measuring the activity of extracellular lactate dehydrogenase (LDH) using the LDH cytotoxicity assay (Thermo Scientific ™ Pierce ™ LDH Cytotoxicity Assay Kit). The procedure was performed according to the manufacturer’s instructions. The method relies on the fact that the cytosolic enzyme LDH is released into the surrounding cell culture media if the cell membrane is damaged. Extracellular LDH undergoes an enzymatic reaction with the assay chemicals culminating in the formation of a red formazan compound, which is measured in a spectrophotometer at 490 nm.

### Quantification of secreted cytokine levels

The levels of different cytokines and growth factors released into the culture media were measured using ELISA (IL-6 and CXCL-8 Standard kits; Biolegend, San Diego, CA) and Luminex analyses (R&D Systems; Minnesota, MN, USA). Luminex analyses were performed to detect the levels of the following 18 cytokines and growth factors: CCL2, CXCL 8/10, interleukin (IL)-1β/1ra/6/8/10/12/15, TNF-α, matrix metalloproteinase (MMP)-1/2/9/10, vascular endothelial growth factor (VEGF), granulocyte–macrophage colony-stimulating factor (GM-CSF) and platelet-derived growth factor (PDGF-AA).

### Reverse transcription PCR (RT-PCR)

The expression levels of selected surface receptors, intracellular signaling molecules and target genes were analyzed with RT-PCR (Table 1). Total RNA was extracted by using GeneJET RNA purification kit (Thermo Scientific) according to the manufacturer’s instructions. The RNA quality was determined using NanoDrop™ 2000 spectrophotometer (Thermo Fisher Scientific, USA). RNA was used for the synthesis of complementary DNA (cDNA) according to the manufacturer’s instructions of maxima first strand cDNA synthesis kit (Thermo Fisher Scientific, Lithuania).

**Table 1:** Sequences of primers that were used for gene expression analysis in HaCaT cells.

Gene	Oligo	Primer sequence
*GAPDH*	Forward	GTCTCCTCTGACTTCAACAGCG
	Reverse	ACCACCCTGTTGCTGTAGCCAA
*TLR2*	Forward	CTTCACTCAGGAGCAGCAAGCA
	Reverse	ACACCAGTGCTGTCCTGTGACA
*TLR4*	Forward	CCCTGAGGCATTTAGGCAGCTA
	Reverse	AGGTAGAGAGGTGGCTTAGGCT
*PAR2*	Forward	CTCCTCTCTGTCATCTGGTTCC
	Reverse	TGCACACTGAGGCAGGTCATGA
*p50*	Forward	GCAGCACTACTTCTTGACCACC
	Reverse	TCTGCTCCTGAGCATTGACGTC
*p65*	Forward	TGAACCGAAACTCTGGCAGCTG
	Reverse	CATCAGCTTGCGAAAAGGAGCC
*c-Jun*	Forward	CCTTGAAAGCTCAGAACTCGGAG
	Reverse	TGCTGCGTTAGCATGAGTTGGC
*c-Fos*	Forward	GCCTCTCTTACTACCACTCACC
	Reverse	AGATGGCAGTGACCGTGGGAAT
*IL-1β*	Forward	CCACAGACCTTCCAGGAGAATG
	Reverse	GTGCAGTTCAGTGATCGTACAGG
*IL-6*	Forward	AGACAGCCACTCACCTCTTCAG
	Reverse	TTCTGCCAGTGCCTCTTTGCTG
*CXCL8*	Forward	GAGAGTGATTGAGAGTGGACCAC
	Reverse	CACAACCCTCTGCACCCAGTTT

SYBR Green (Maxima® SYBR Green/ROX qPCR Master Mix, Thermo Fisher Scientific, Lithuania) was performed according to the thermal cycling conditions suggested by the manufacturers. Briefly, the thermal profile consisted of an initial denaturation at 50 °C for 10 min, followed by 40 cycles of denaturation at 95 °C for 15 s, annealing /extension at 60 °C for 60 s. Finally, melting curve analysis was performed at 95 °C for 20 s after every experiment. Cycle Threshold (CT) values were determined using ABI 7900HT Fast Real-Time PCR system (Applied Biosystems) and gene expression was normalized against GAPDH. Relative quantification of gene expression was determined by using the ΔΔCt method and fold changes were generated by using the Eq. 2^ΔΔCt^^20^.

### IncuCyte live imaging system

IncuCyte S3 Live Cell Analysis System was used to visualize and monitor bacterial growth and cell death, in the presence of PLNC8 αβ and gentamicin, either alone or in combination. The cells were seeded in 96 well plates (2 × 10^4^ cells/well) in complete cell culture media composed of phenol red-free DMEM supplemented with 10% FBS, and incubated overnight at (37 °C, 5% CO_2_). Cells were either left untreated, infected with *S. aureus* (MOI 1, MOI 0.1) expressing green fluorescent protein (GFP), and exposed to PLNC8 αβ (6.25, 12.5 and 25 µM) or gentamicin (1 and 0.1 µg/ml) alone, or a combination of both PLNC8 αβ and gentamicin. Bacterial growth and cell death were monitored for 93 h using a 20 × objective lens. *S. aureus* growth was monitored by detecting the fluorescence intensity of GFP and the number of dead cells were registered by detecting the fluorescence of Cytotox Reagent DRAQ7 (BD Pharmingen™) at 1 μM concentration. Videos and photos for the whole scan time were acquired and analysed using IncuCyte S3 Live Cell Analyses System (Sartorius AG, Göttingen, Germany). Flourescence intensity was expressed as arbitrary units (AU) per well.

### Statistical analysis

All data were analyzed using GraphPad Prism 5.0 (GraphPad Software, La Jolla, CA, USA). One-way ANOVA with Sidak’s multiple comparison test was used for the comparisons between the different treatments.

## Results

### Antimicrobial activity of PLNC8 αβ on S. aureus

**Figure 1:**
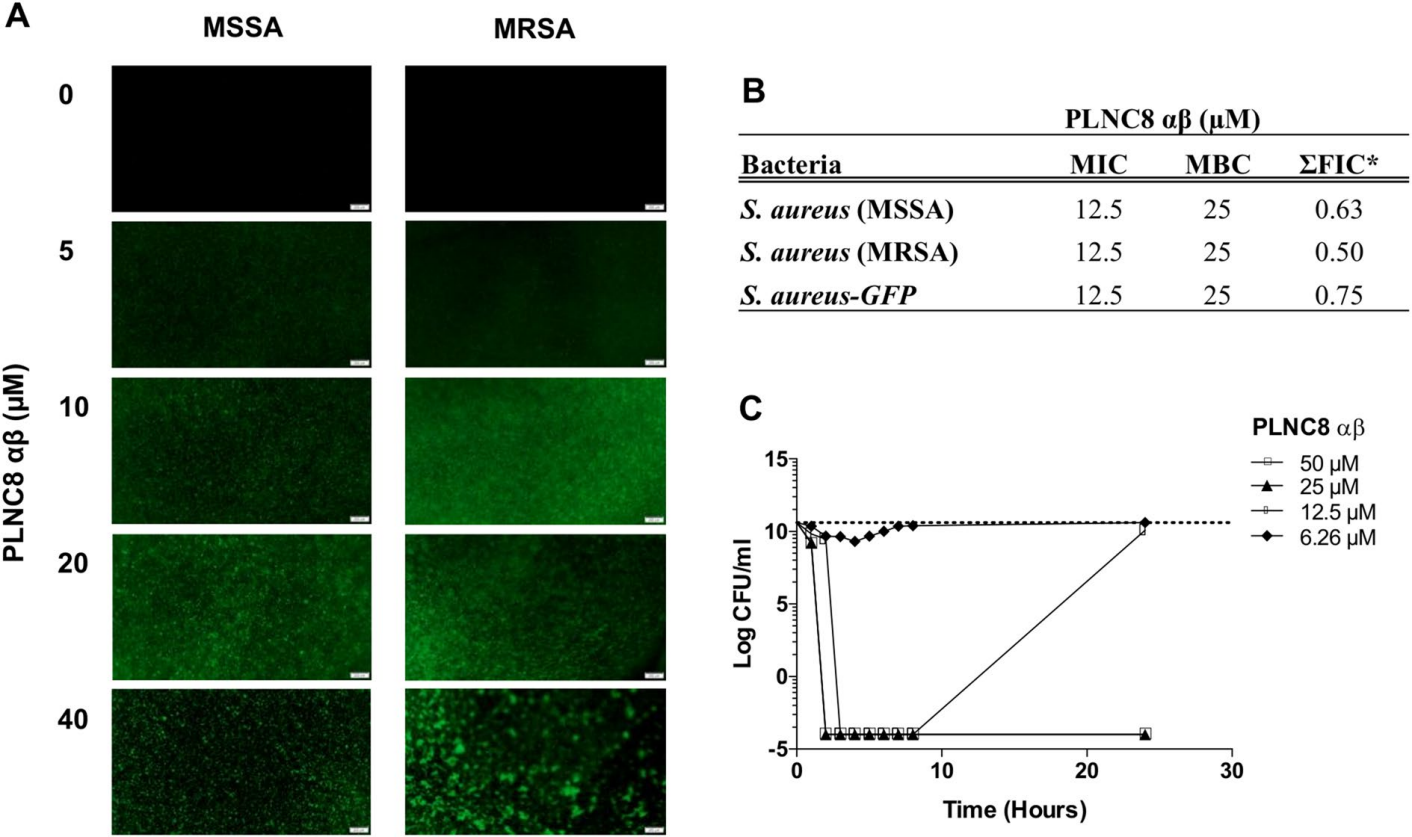
PLNC8 αβ causes rapid permeabilization of *S. aureus*, independent of their susceptibility towards methicillin. (**A**) Permeabilization of *S. aureus* in a dose-dependent manner by PLNC8 αβ after exposure for 5 min, as indicated by Sytox green staining. (**B**) MIC and MBC values for MSSA and MRSA are indicated. *ΣFIC values of PLNC8 αβ in combination with Gentamicin were determined using checkerboard analysis. (**C**) Time-kill assay. The antibacterial effect of PLNC8 αβ at different concentrations over a period of 24 h shows the effects of the peptide in a dose-dependent manner. The y-axis indicates the viable count (CFU/mL) of *S. aureus* at certain timepoints.

Bacterial permeabilization by PLNC8 αβ was studied by using the fluorescent nucleic acid stain SYTOX™ Green. PLNC8 αβ was found to permeabilize *S. aureus* already after 5 min in a dose-dependent manner (Fig. 1A), as indicated by the increased fluorescence of Sytox green. Furthermore, high PLNC8 αβ concentrations caused more prominent aggregation of the bacteria, indicating efficient permeabilization and secretion of bacterial content. The antimicrobial effect of PLNC8 αβ on *S. aureus* was further determined using the broth microdilution method. PLNC8 αβ was found to target MSSA and MRSA with similar efficacy, thus irrespective of their susceptibility towards methicillin (Fig. 1B). MIC and MBC values was determined to be 12.5 µM and 25 µM, respectively. To complement these data, the bactericidal activity of PLNC8 αβ was evaluated over time, using a time-kill assay. The bactericidal effect of PLNC8 αβ was rapid, and a final concentration of 25 and 50 µM completely eliminated the bacteria after 2 h (Fig. 1C). At lower peptide concentrations, the bactericidal effect was evident at early time points, by substantially reducing the CFU numbers, but the bacteria could rapidly recover and grow when the peptides have been consumed.

### PLNC8 αβ counteracts S. aureus-mediated cytotoxicity and inflammatory responses

**Figure 2:**
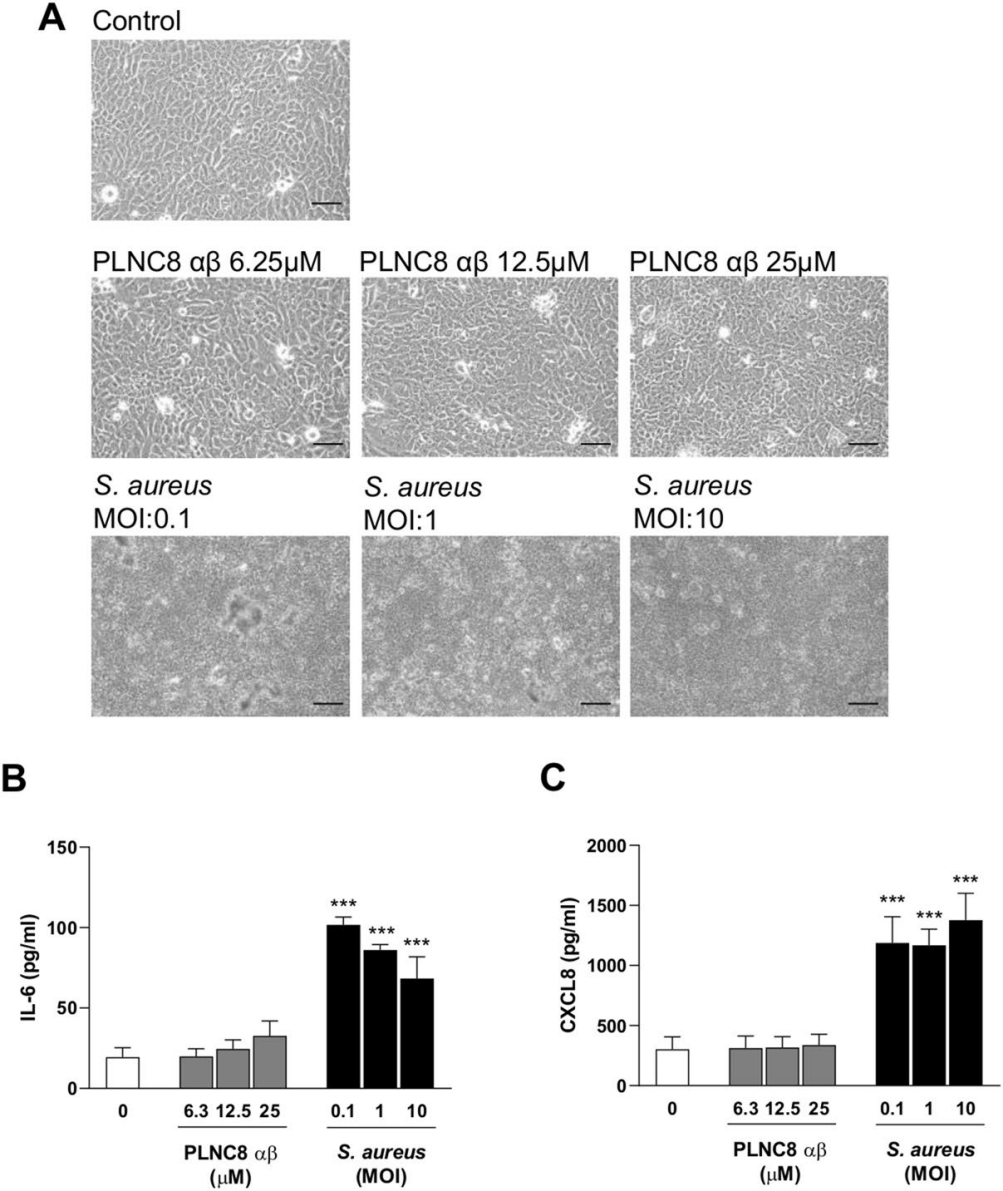
PLNC8 αβ shows no effect on viability and the inflammatory responses of human keratinocytes. (**A**) Representative images of HaCaT cells that were exposed to different concentrations of PLNC8 αβ or *S. aureus* for 24 h, scale bar is 50 µm. (**B**) *S. aureus*, but not PLNC8 αβ, increased the secretion of (**C**) IL-6 and (**D**) CXCL8. ***p < 0.001 (One-way ANOVA with Sidak’s post-hoc test).

**Figure 3:**
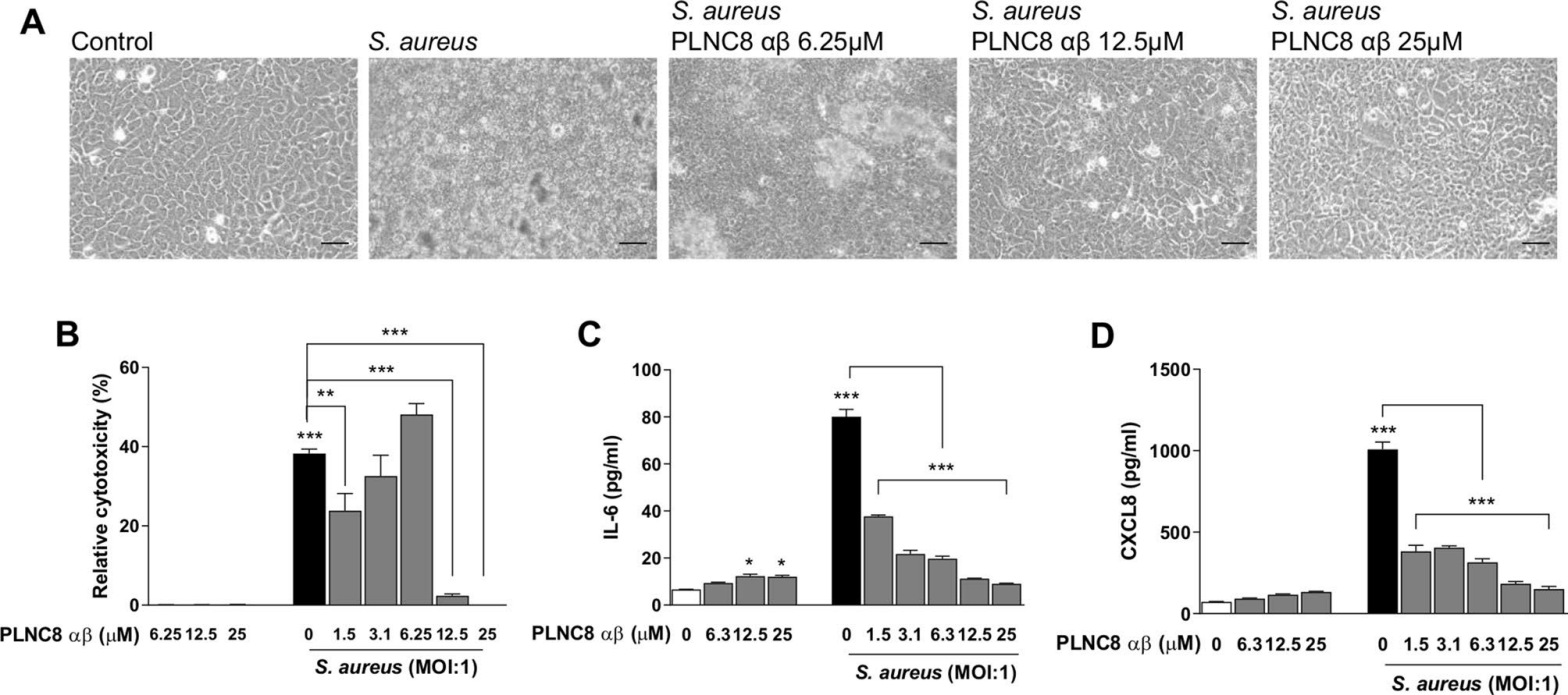
PLNC8 αβ reduces inflammatory responses of *S.aureus*-infected human keratinocytes. (**A**) Representative images of HaCaT cells infected with *S. aureus* for 1 h, followed by addition of PLNC8 αβ for 24 h, scale bar is 50 µm. (**B**) PLNC8 αβ alone is not cytotoxic, and higher concentrations of the peptide substantially prevented *S. aureus*-mediated cytotoxicity. The increased secretion of (**C**) IL-6 and (**D**) CXCL8 by *S. aureus* was significantly reduced by the peptides. ***p < 0.001 (One-way ANOVA with Sidak’s post-hoc test).

The effects of PLNC8 αβ and *S. aureus* on eukaryotic cells were studied by using the human keratinocyte cell line HaCaT. The cells were either exposed to PLNC8 αβ or infected with *S. aureus* alone at different concentrations and MOIs for 24 h. Microscopic analysis showed that the cells retained normal morphology after exposure to PLNC8 αβ, with no signs of cell death. In contrast to this, *S. aureus* had severe cytotoxic effects on the keratinocytes, irrespective of the initial MOI (Fig. 2A). Furthermore, PLNC8 αβ alone did not affect the expression of the inflammatory mediators IL-6 and CXCL8, while *S. aureus* caused a significant upregulation of both (Fig. 2B–C). These results encouraged the design of additional experiments with the aim to determine the effects of PLNC8 αβ on *S. aureus*-infected cells. The cells were infected for 1 h with *S. aureus* prior to addition of a single dose, consisting of different concentrations of PLNC8 αβ, and was then incubated for 24 h. The peptides suppressed bacterial growth and preserved in a dose-dependent manner normal morphology and viability of keratinocytes, compared to infected cells (Fig. 3A). PLNC8 αβ alone showed no cytotoxic effects on the keratinocytes, as determined by measuring LDH activity (Fig. 3B). Furthermore, the peptides efficiently suppressed *S. aureus*-mediated cytotoxicity at the two highest doses (12.5 and 25 µM). However, the lower doses ranging from 1.5 to 6.25 µM were not sufficient to counteract the *S. aureus*-mediated cytotoxicity. Indeed, the lower doses showed an increasing trend in cytotoxicity, although no significant differences were found compared to the control. The cytokines IL-6 and CXCL8 were quantified as markers of inflammation. *S. aureus* induced high levels of both IL-6 (Fig. 3C) and CXCL8 (Fig. 3D), while the peptides by themselves did not alter their expression to the same extent as the infected cells. PLNC8 αβ suppressed the inflammatory responses induced by *S. aureus* in a dose-dependent manner. Interestingly, a final PLNC8 αβ concentration of 12.5 µM and 25 µM lowered both IL-6 and CXCL8 close to basal levels.

**Figure 4:**
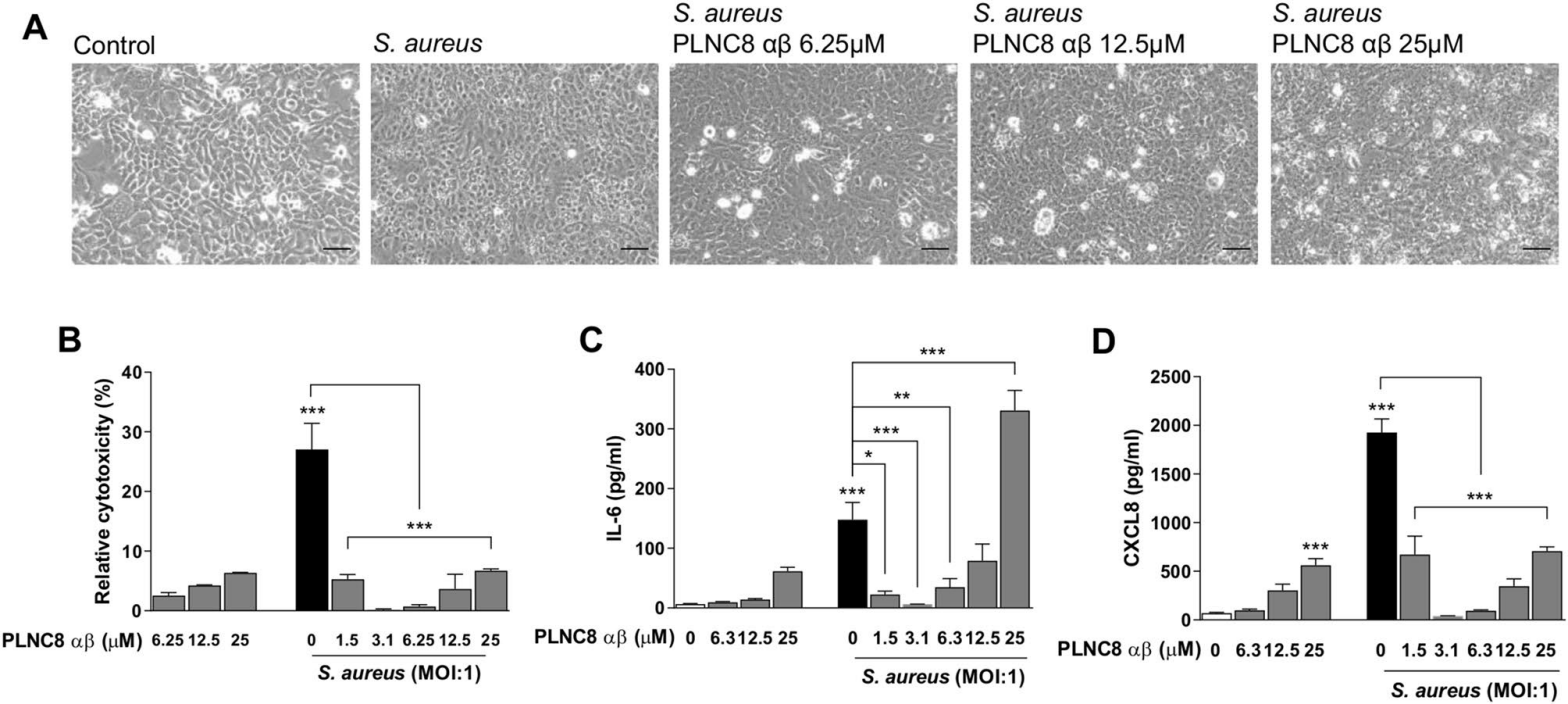
PLNC8 αβ antagonizes *S. aureus*-induced cytotoxicity and modulates the inflammatory responses of human keratinocytes. (**A**) Representative images of HaCaT cells after infection with *S. aureus* for 1 h followed by addition of PLNC8 αβ for 6 h, scale bar is 50 µm. (**B**) PLNC8 αβ antagonized *S. aureus*-mediated cytotoxicity, which was determined by measuring LDH activity, and promoted cell viability. The secretion of (**C**) IL-6 and (**D**) CXCL8 was significantly reduced by the peptides, except the highest dose (25 µM), which increased IL-6 secretion. *p < 0.05; **p < 0.01; ***p < 0.001 (One-way ANOVA with Sidak’s post-hoc test).

Early effects of the peptides and the bacteria on keratinocytes and their inflammatory responses were studied by infecting HaCaT cells for 1 h followed by addition of PLNC8 αβ for 6 h (Fig. 4). Microscopy images showed that the cells were affected by the bacteria during this short-time infection, as they appeared round shaped compared to untreated cells. The rapid antimicrobial activity of PLNC8 αβ had beneficial effects by markedly suppressing bacterial growth, as the keratinocytes retained normal cell morphology (Fig. 4A). PLNC8 αβ alone showed minor cytotoxic (Fig. 4B) and inflammatory effects (Fig. 4C–D), when compared to the infected cells. Addition of PLNC8 αβ to the infected cells was found to significantly reduce the cytotoxic effect of *S. aureus* (Fig. 4B) on human keratinocytes and their inflammatory responses, IL-6 (Fig. 4C) and CXCL-8 (Fig. 4D). The keratinocyte inflammatory response regarding IL-6 and CXCL-8 at the two lower concentrations (6.25 µM and 12.5 µM) was significantly reduced compared to the infected cells. However, the highest concentration of 25 µM showed a significant increase in IL-6 (Fig. 4C) but not CXCL-8 (Fig. 4D). Addition of 25 µM Peptide concentration alone was found to acutely increase the level of inflammatory cytokines (IL-6 and CXCL-8) secretion from keratinocytes. Moreover, 25 µM PLNC8 αβ was found to induce release of IL-6 from infected keratinocytes which can be due to the effectiveness of this dose in the lysis of bacteria and subsequent release of the PAMPS that lead to stimulation of the keratinocyte’s inflammatory response.

Furthermore, gene expression analysis was performed to investigate the involvement of certain receptors and intracellular signalling molecules following infection of keratinocytes with *S. aureus*, in the presence or absence of PLNC8 αβ, and to verify the results of IL-6 and CXCL8 on the mRNA level. Exposure of cells to the peptides alone induced, in a dose-dependent manner, gene expression of all target genes, except *p50* and *p65* (Table 2A). Interestingly, *c-Jun* was highly upregulated by PLNC8 αβ at all concentrations. However, the *S. aureus*-induced gene expression was more pronounced, particularly *TLR2*, *TLR4*, *c-Fos* and the inflammatory mediators *IL-1β*, *IL-6* and *CXCL8*. Addition of PLNC8 αβ effectively counteracted the expression of all target genes induced by the bacteria. The role of *TLR4* and *c-Jun* in the initiation of inflammation of keratinocytes is obvious since their gene expression levels remained elevated even after addition of the peptides. The results further confirm our previous findings in this study of low PLNC8 αβ concentrations (3.1 µM and 6.25 µM) being optimal doses at counteracting the effects of *S. aureus* in the early phases of infection.

**﻿Table 2:** PLNC8 αβ modulates gene expression of important receptors and intracellular signaling transducers in human keratinocytes.

*(A)*										
					*S. aureus* (MOI:1)					
PLNC8 αβ (µM)										
Gene	0	6.25	12.5	25	0	1.5	3.1	6.25	12.5	25
*TLR2*	1.0 ± 0.2	1.5 ± 0.1	1.5 ± 0.1	2.0 ± 0.2*	2.5 ± 0.2*	1.3 ± 0.1^#^	1.0 ± 0.5^#^	1.3 ± 0.4^#^	2.0 ± 0.2*	1.8 ± 0.3*
*TLR4*	1.0 ± 0.2	2.1 ± 0.6	1.7 ± 0.1	6.5 ± 0.4*	9.3 ± 0.6*	1.9 ± 0.4^#^	1.8 ± 0.5^#^	1.3 ± 0.5^#^	3.6 ± 0.5*^#^	8.6 ± 1.9*
*PAR2*	1.0 ± 0.1	1.4 ± 0.2	2.1 ± 0.2*	5.4 ± 0.5*	2.0 ± 0.1	1.9 ± 0.5	1.0 ± 0.1	1.3 ± 0.6	2.8 ± 0.5*	5.7 ± 0.8*^#^
*p50*	1.0 ± 0.1	0.9 ± 0.2	1.1 ± 0.2	1.1 ± 0.2	1.3 ± 0.2	0.9 ± 0.1^#^	0.8 ± 0.1^#^	0.8 ± 0.1^#^	0.9 ± 0.1^#^	1.2 ± 0.1
*p65*	1.0 ± 0.1	1.0 ± 0.1	1.0 ± 0.1	1.0 ± 0.1	1.0 ± 0.1	1.0 ± 0.1	1.0 ± 0.1	0.8 ± 0.2	0.9 ± 0.2	1.0 ± 0.1
*c-jJn*	1.0 ± 0.1	8.3 ± 2.1	11 ± 3.4*	21 ± 0.9*	17 ± 11*	4.5 ± 1.0^#^	6.7 ± 2.5	0.8 ± 0.3	0.9 ± 0.3*	50 ± 6.9*^#^
*c-Fos*	1.0 ± 0.1	1.3 ± 0.2	2.1 ± 0.4	4.6 ± 0.5	51 ± 42*	7.6 ± 0.9^#^	1.2 ± 0.2^#^	0.8 ± 0.4^#^	0.9 ± 0.4^#^	50 ± 13*
*IL-1β*	1.0 ± 0.2	0.8 ± 0.1	0.8 ± 0.2	1.3 ± 0.1	34 ± 4.1*	6.3 ± 1.3*^#^	1.2 ± 0.1^#^	0.8 ± 0.1^#^	0.9 ± 0.1^#^	1.6 ± 0.6^#^
*IL-6*	1.0 ± 0.2	1.9 ± 0.4	5.9 ± 5.7	8.3 ± 2.8	259 ± 103*	16 ± 1.1^#^	3.4 ± 0.7^#^	3.1 ± 1.3^#^	8.6 ± 2.2^#^	83 ± 86*^#^
*CXCL8*	1.0 ± 0.2	2.8 ± 1.1	5.8 ± 3.2	23 ± 10	221 ± 80*	60 ± 11^#^	3.4 ± 0.6^#^	3.7 ± 0.9^#^	13 ± 5.9^#^	67 ± 64*^#^

*(B)*										
Gene	0	6.25	12.5	25						
*TLR2*	1.0 ± 0.3	1.0 ± 0.1	1.0 ± 0.3	1.2 ± 0.5						
*TLR4*	1.0 ± 0.2	1.1 ± 0.1	1.0 ± 0.1	0.8 ± 0.2						
*PAR2*	1.0 ± 0.1	1.1 ± 0.1	1.1 ± 0.1	1.1 ± 0.2						
*p50*	1.0 ± 0.1	1.3 ± 0.7	1.1 ± 0.1	0.9 ± 0.3						
*p65*	1.0 ± 0.1	0.8 ± 0.1	0.8 ± 0.1	1.0 ± 0.1						
*c-Jun*	1.0 ± 0.4	1.4 ± 0.2	1.6 ± 0.2	1.4 ± 0.2						
*c-Fos*	1.0 ± 0.1	1.2 ± 0.2	1.2 ± 0.2	0.8 ± 0.4						
*IL-1β*	1.1 ± 0.4	0.8 ± 0.1	0.7 ± 0.1	0.6 ± 0.1						
*IL-6*	1.0 ± 0.2	1.1 ± 0.1	1.2 ± 0.4	0.6 ± 0.2						
*CXCl8*	1.0 ± 0.2	1.1 ± 0.2	1.0 ± 0.4	1.0 ± 0.6						

Gene expression analysis was performed after infection of human keratinocytes with *S. aureus* for 1 h, followed by addition of PLNC8 αβ at the indicated concentrations for (**A**) 6 h or (**B**) 24 h. The results show that PLNC8 αβ modulates cellular responses through the receptors *TLR2*, *TLR4* and *PAR2*, and the intracellular signaling transducers *c-Jun* and *c-Fos*, suggesting a role for the transcription factor AP-1 in the early inflammatory responses and cell survival. Decimals were removed for number > 10. One-way ANOVA with Sidak’s multiple comparison test, p-values of < 0.05 are shown, compared to the *negative control (without bacteria or peptides) and #positive control (*S. aureus*-infected cells without PLNC8 αβ).

The induction of gene expression by the peptides alone encouraged us to investigate whether these effects were acute or persistent responses by the cells. Analysis of gene expression after 24 h of exposure to PLNC8 αβ showed no effects (Table 2B), indicating that the effects of the peptides on the keratinocytes are short-lived, and that the cellular response is acute.

Furthermore, multiplex analysis was used to detect the release of an array of cytokines, MMPs and growth factors after 6 h and 24 h of infection with *S. aureus* and exposure to PLNC8 αβ. The release of these factors in response to PLNC8 αβ was more evident at 6 h (Table 3A) compared to 24 h (Table 3B). *S. aureus* upregulated the expression of cytokines, MMPs and growth factors and the presence of PLNC8 αβ efficiently altered these levels. While the peptides decreased *S. aureus*-induced cytokine and MMP expression, interestingly, PDGF-AA was further induced by PLNC8 αβ after 6 h. The levels of all factors were generally low after 24 h of infection with *S. aureus*, which is probably a consequence of cells no longer being viable. Our theory that PLNC8 αβ targets the bacteria and thus contributes towards maintaining cellular viability is reflected by elevated levels of MMP1, MMP2, MMP9, MMP10, VEGF and PDGF-AA by PLNC8 αβ in a dose-dependent manner (Table 3B).

**﻿Table 3:** Differential release of cytokines and growth factors by keratinocytes in response to PLNC8 αβ.

**(A)**										
					*S. aureus* (MOI:1)					
PLNC8 αβ (µM)										
Protein (pg/ml)	0	6.25	12.5	25	0	1.5	3.1	6.25	12.5	25
IL-6	1.3 ± 0.1	1.4 ± 0.1	1.5 ± 0.1	12 ± 0.6*	11 ± 3.1*	1.8 ± 0.1^#^	1.4 ± 0.2^#^	1.6 ± 0.1^#^	3.8 ± 1.0^#^	49 ± 1.2*^#^
CXCL8	9.2 ± 0.3	21 ± 1.1	30 ± 2.4	837 ± 34*	1485 ± 342*	108 ± 29^#^	28 ± 2.1^#^	47 ± 2.9^#^	230 ± 57^#^	1442 ± 357*
IL-1RA	1207 ± 82	1719 ± 760	1561 ± 172	3918 ± 366*	2439 ± 337*	1421 ± 105^#^	782 ± 73^#^	976 ± 55^#^	1514 ± 77^#^	6689 ± 230*^#^
MMP1	654 ± 18	789 ± 118	789 ± 25	898 ± 56*	713 ± 114	713 ± 27	496 ± 85	580 ± 67	642 ± 75	678 ± 18
MMP2	2097 ± 119	2971 ± 375	3307 ± 241*	2868 ± 85	2888 ± 492	3162 ± 124*	2266 ± 459^#^	2399 ± 298	2911 ± 502	2657 ± 164
MMP9	34 ± 4.7	66 ± 3.4	75 ± 5.7*	189 ± 8.9*	43 ± 8.9	32 ± 4.9	43 ± 5.4	55 ± 1.9	100 ± 4.4*^#^	244 ± 45*^#^
MMP10	459 ± 21	654 ± 103*	692 ± 40*	977 ± 12*	523 ± 94	533 ± 27	365 ± 64	425 ± 49	547 ± 82	704 ± 39*^#^
MCP1	47 ± 4.7	58 ± 3.8	51 ± 4.2	132 ± 5.6	1681 ± 118*	200 ± 36*^#^	76 ± 1.7^#^	130 ± 10^#^	286 ± 9.4*^#^	255 ± 45*^#^
GM-CSF	1.1 ± 0.3	2.0 ± 0.3	1.0 ± 0.3	2.5 ± 0.1	7.2 ± 1.5*	1.2 ± 0.6^#^	1.1 ± 0.4^#^	0.9 ± 0.4^#^	1.6 ± 0.2^#^	6.1 ± 1.4*
VEGF	40 ± 2.2	63 ± 0.9*	62 ± 1.9*	158 ± 9.1*	106 ± 13*	49 ± 4.9^#^	42 ± 2.6^#^	67 ± 2.6*^#^	76 ± 2.3*^#^	165 ± 10*^#^
PDGF-AA	84 ± 3.7	243 ± 4.5*	258 ± 6.9*	254 ± 8.0*	92 ± 8.0	138 ± 4.2*^#^	142 ± 7.5*^#^	202 ± 14*^#^	219 ± 5.9*^#^	177 ± 7.3*^#^

**(B)**										
Protein (pg/ml)	0	6.25	12.5	25	0	1.5	3.1	6.25	12.5	25
IL-6	0.6 ± 0.2	1.2 ± 0.2	1.4 ± 0.2	1.5 ± 0.1	13 ± 2.0*	6.5 ± 0.4*^#^	2.9 ± 0.1*^#^	3.2 ± 0.5*^#^	1.2 ± 0.4^#^	1.1 ± 0.2^#^
CXCL8	104 ± 3.6	125 ± 8.2	142 ± 10	192 ± 3.4	504 ± 82*	297 ± 18*^#^	514 ± 47*	319 ± 40*^#^	154 ± 12^#^	184 ± 24^#^
IL-1RA	1090 ± 179	884 ± 81	899 ± 69	792 ± 14	> 6820*	> 6820*	> 6820*	> 6820*	3598 ± 1391*^#^	1228 ± 219^#^
MMP1	3217 ± 13	3403 ± 150	3777 ± 156*	3846 ± 126*	1412 ± 182*	1636 ± 132*	1541 ± 56*	2389 ± 169*^#^	3177 ± 151^#^	3445 ± 69^#^
MMP2	5694 ± 221	4519 ± 232*	4029 ± 283*	3127 ± 104*	633 ± 94*	1581 ± 228*^#^	1818 ± 135*^#^	2066 ± 198*^#^	3306 ± 325*^#^	3056 ± 202*^#^
MMP9	42 ± 2.9	43 ± 4.3	53 ± 1.9	84 ± 4.7*	57 ± 6.4*	81 ± 2.9*^#^	90 ± 7.1*^#^	79 ± 6.9*^#^	72 ± 4.9*^#^	105 ± 2.5*^#^
MMP10	3229 ± 134	2846 ± 197	3540 ± 241	4379 ± 175*	1941 ± 111*	2010 ± 40*	2149 ± 154*	2753 ± 202^#^	3789 ± 168^#^	5086 ± 426*^#^
MCP1	22 ± 1.4	19 ± 1.0	20 ± 1.0	19 ± 1.0	18 ± 0.5*	16 ± 1.4*	16 ± 1.3*	17 ± 1.3*	19 ± 0.7*	19 ± 1.8
GM-CSF	7.7 ± 0.4	9.8 ± 0.4	12 ± 1.0*	13 ± 0.9*	41 ± 1.4*	22 ± 2.5*^#^	20 ± 2.1*^#^	20 ± 1.2*^#^	8.6 ± 2.1^#^	9.9 ± 0.7^#^
VEGF	641 ± 4.3	692 ± 34	757 ± 33*	701 ± 33	504 ± 57*	683 ± 9.0^#^	508 ± 29*	612 ± 16^#^	689 ± 33^#^	655 ± 46^#^
PDGF-AA	238 ± 5.5	291 ± 14*	337 ± 10*	318 ± 11*	70 ± 5.3*	114 ± 9.9*^#^	138 ± 6.7*^#^	157 ± 8.0*^#^	269 ± 15*^#^	281 ± 8.5*^#^

Multiplex analysis of human keratinocytes after infection with *S. aureus* for 1 h followed by addition of the indicated concentrations of PLNC8 αβ for (**A**) 6 h or (**B**) 24 h. PLNC8 αβ alone induced the release of different keratinocyte factors, while low concentration of PLNC8 αβ significantly suppressed *S. aureus*-induced inflammatory mediators. Decimals are removed for number > 10. One-way ANOVA with Sidak’s multiple comparison test, p-values of < 0.05 are shown, compared to the *negative control (without bacteria or peptides) and #positive control (*S. aureus*-infected cells without PLNC8 αβ).

**Figure 5:**
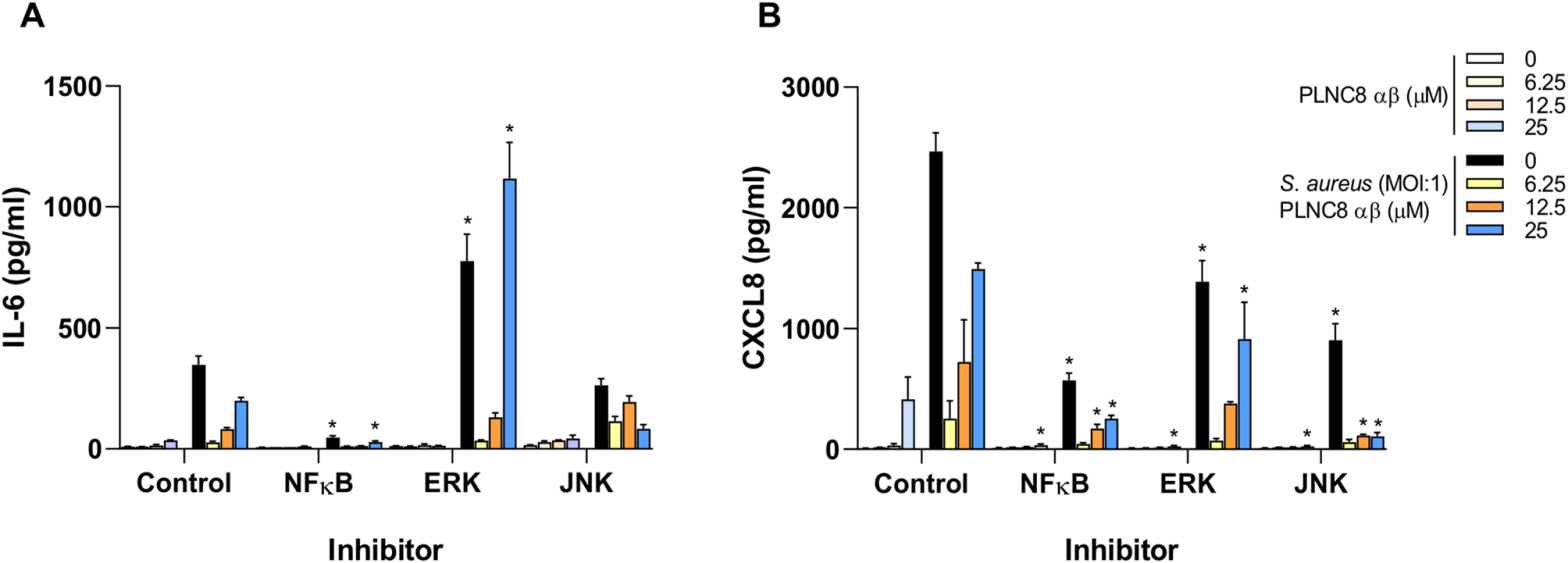
NFκB is an important regulator of inflammatory responses in human keratinocytes. Human keratinocytes (HaCaT) were treated with the indicated inhibitors for 2 h, followed by infection with *S. aureus* for 1 h and then addition of PLNC8 αβ for 6 h. (**A**) IL-6 and (**B**) CXCL8 secretion was significantly reduced, primarily after inhibition of NFκB and JNK, but not ERK. *P*-values of < 0.05 are shown (One-way ANOVA with Sidak’s post-hoc test), compared to their counterpart in the uninhibited group.

The involvement of the specific transcription factors, NFκB, ERK and JNK, in the initiation of inflammatory processes of keratinocytes was investigated by using specific inhibitors. The results confirmed the involvement of c-Jun in IL-6 (Fig. 5A) and CXCL8 (Fig. 5B) expression, since inhibition of JNK significantly reduced the levels of these inflammatory cytokines. The transcription factor NFκB was shown to play a major role in the induction of inflammatory responses of human keratinocytes. Accordingly, inhibition of NFκB and JNK also affected IL-6 and CXCL8 release in response to PLNC8 αβ, with or without bacteria. Inhibition of ERK upregulated IL-6 and suppressed CXCL8 compared to the control.

### Additive or synergistic antimicrobial activity of PLNC8 αβ and gentamicin

**Figure 6:**
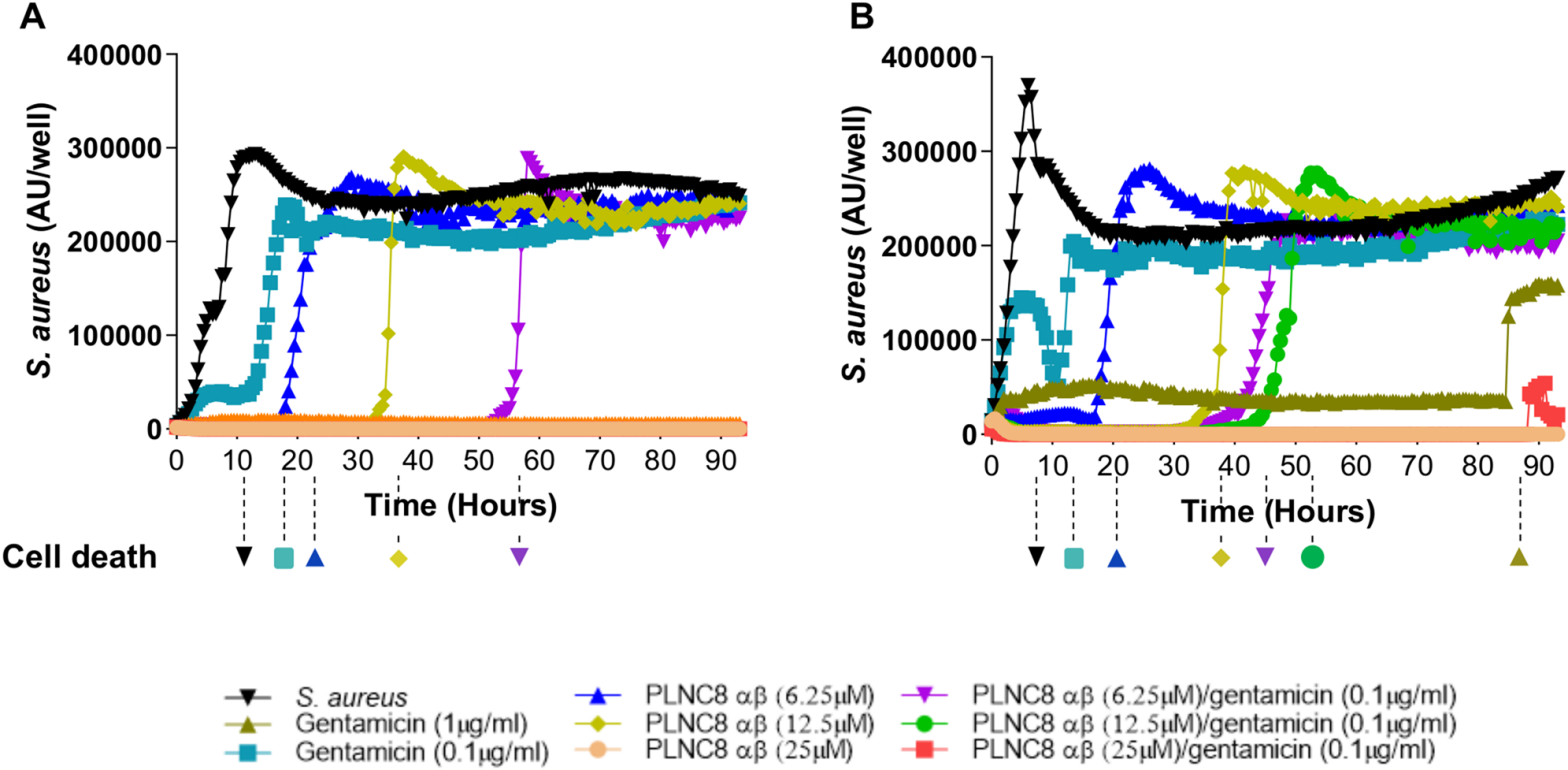
PLNC8 αβ inhibits *S. aureus* growth and bacteria-induced cytotoxicity of human keratinocytes. HaCaT cells were infected with MSSA, in the presence or absence of PLNC8 αβ for 93 h. PLNC8 αβ inhibited *S. aureus* growth at (**A**) MOI:0.1 and (**B**) MOI:1 in a dose-dependent manner for at least 93 h, and markedly enhanced the effect of low dose gentamicin. *S. aureus* caused cell death after 11 h (MOI:0.1) and 7 h (MOI:1), as indicated by the dotted lines and markers below each graph. PLNC8 αβ efficiently counteracted this effect in a dose-dependent manner. The combination of PLNC8 αβ with gentamicin synergistically inhibited *S. aureus* growth and counteracted bacteria-induced cell death. The y-axis represents fluorescence intensity expressed as arbitrary units (AU/well).

IncuCyte live-cell imaging and associated analysis system was used to monitor the growth rate of *S. aureus* and their cytotoxic effects on HaCaT cells, in the presence or absence of PLNC8 αβ and gentamicin, either alone or in combination. *S. aureus* (MOI:0.1) growth was rapid and the bacteria were cytotoxic shortly after their density reached a plateau at ~ 11 h (Fig. 6A). A low concentration of gentamicin transiently suppressed bacterial growth and prevented cell death up to 16 h, while a higher concentration was effective throughout the entire scan period (93 h). PLNC8 αβ, at a final concentration of 6.25, 12.5 and 25 µM, suppressed bacterial growth and protected the cells in a dose-dependent manner, up to 23 h, 36 h and > 93 h, respectively (Fig. 6A). Interestingly, a combination of 6.25 µM of PLNC8 αβ and 0.1 µg/ml gentamicin efficiently inhibited the bacteria and their cytotoxicity for 57 h, while a combination of 12.5 µM or 25 µM PLNC8 αβ with 0.1 µg/ml gentamicin effectively eliminated all the bacteria over the whole test period. Similar trends were observed when the bacterial concentration was increased tenfold to MOI:1 (Fig. 6B). Checkerboard assays revealed an additive effect of gentamicin and PLNC8 αβ for MSSA and MSSA-GFP, with a ΣFIC of 0.625 and 0.75 respectively. For MRSA the results implied a synergistic relationship, with a ΣFIC of 0.5 (Fig. 1B).

## Discussion

Antibiotic resistance is a constantly growing global threat, and *S. aureus* has been identified by the World Health Organization (WHO) as one of the priority pathogens for which novel antimicrobials are urgently needed. *Staphylococcus spp*. are the primary pathogens in skin and soft tissue infections^21^. *S. aureus* also contributes to several severe infections in humans^22^ and treatment may become complicated due to the resistance against the wide array of antibiotics that this pathogen has acquired^23^. Development of non-conventional therapies would therefore be advantageous, and bacteriocins represent an interesting novel route as the next generation of antibacterial agents^17,24^. We have previously reported that PLNC8 αβ has antimicrobial activity against the gram negative bacterium *P. gingivalis*^25^ and recently we found that PlnA, E, F, J, K, NC8 αβ of *Lactobacillus plantarum* are potent antimicrobials against *Staphylococcus* species^19,26^. The antimicrobial activity of PLNC8 αβ against *S. aureus*, both antibiotic-sensitive and resistant strains, was investigated to verify our previous findings^26^. Bacterial permeabilization by PLNC8 αβ was recorded within minutes, and the inhibitory and bactericidal concentrations of PLNC8 αβ were found to be the same for both strains. These results indicate that the peptides have high selectivity toward *Staphylococcus spp*., most likely mediated by an electrostatic interaction between positively charged peptides and negatively charged phospholipids of the bacterial cell membrane^26^. The aim of the present study was to evaluate the role of PLNC8 αβ as antimicrobial and anti-inflammatory agent on human keratinocytes infected with *S. aureus*.

Evaluation of eventual toxic effects and inflammatory reactions of host cells caused by PLNC8 αβ is necessary for future applications of the peptides as therapeutic agents against bacterial infections^27^. Keratinocytes are the main cell type in the epidermis, which is the outermost skin layer constituting the first line of host defense by creating a physical and chemical barrier between the body and external microorganisms^28^. In this study, we found that exposure of human keratinocytes to PLNC8 αβ did not affect cell viability, and short-term exposure of the peptide upregulated several cytokines, MMPs and growth factors at the gene and protein level, however after long-term exposure protein levels eventually returned to basal levels. These early effects may be due to the short half-life of the peptides in the presence of proteinases, and in support we have previously shown that the L-enantiomer of PLNC8 αβ is completely degraded by trypsin after 16 h^26^. Release of trypsin, a keratinocyte endogenous protease, was reported to be induced by *S. aureus* infection^29^.

Gene expression analysis showed that PLNC8 αβ enhances the transcription of all the investigated genes, except *p50* and *p65*. Increased expression of PRRs (*TLR2*, *TLR4*, *PAR2*), intracellular MAPK signaling molecules (*c-Jun*, *c-Fos*) and inflammatory mediators (*IL-1β*, *IL-6*, *CXCL8*) may ultimately improve pathogen recognition, cell survival and cellular communication, respectively, possibly leading to an increased elimination of invading pathogens. Indeed, activation of TLR2 by peptidoglycan improves the tight junction barrier of human keratinocytes, which is essential in preventing bacterial invasion^30^. Menzies and colleagues^31^ emphasized for the recognition of *S. aureus* the importance of keratinocyte TLR2, which activates the MAPK signaling pathway, suggesting a role of c-Fos and c-Jun in the early inflammatory responses and cell survival. This in turn infers the involvement of activator protein 1 (AP-1), since nuclear translocation of AP-1 was subsequently followed by induction of CXCL8 and the antimicrobial peptide human β-defensin 3. However, increased TLR2 expression can have negative effects on diabetic wounds, as this receptor contributes to prolonged inflammation that impairs wound healing^32^. From a clinical perspective, it could be advantageous to induce an acute cell response of low levels of inflammatory mediators, including cytokines, chemokines, MMPs and growth factors, to promote cellular communication, enhance pathogen elimination and promote healing of acute wounds. MMPs are expressed at the edges of a wound by keratinocytes and can be induced by cytokines and growth factors^33^, reflecting the importance of cellular communication via inflammatory mediators and the role of MMPs in wound healing processes^34^. Adequate expression of these mediators is critical since inhibition or overexpression may impair healing and induce diseases^35^.

Interestingly, addition of PLNC8 αβ significantly suppressed *S. aureus*-mediated inflammatory responses to basal levels and promoted cell viability. These results indicate that the peptides display higher preference towards the bacteria than they do to eukaryotic cells^36^, which is most probably due to the biophysical properties of the peptides (cationic, amphipathic) that primarily target negatively charged microbial membranes, circumventing the zwitterionic eukaryotic cell membranes^26,37^. Similar trends were observed for all the analyzed genes and proteins, except for PDGF-AA, which was suppressed by *S. aureus*, an effect that was counteracted by PLNC8 αβ. PDGF is an important growth factor with its potent mitogenic and chemotactic properties^38^, and has been approved for clinical use to accelerate wound healing of complicated disorders, e.g. ulcers in diabetic patients^39,40^. This further demonstrates the potential beneficial effects of PLNC αβ on superficial skin infections.

The gene- and protein expression differed following infection of keratinocytes with *S. aureus* and in the presence of PLNC8 αβ after 6 h and 24 h. PLNC8 αβ changed the expression/release patterns of inflammatory mediators in a time- and dose-dependent way. The antimicrobial activity of PLNC8 αβ is rapid (min) and causes significant adverse antimicrobial effects on staphylococci, including thickening of the cell wall, detachment of the cell membrane and release of substantial amounts of intracellular content^26^. This may lead to exposure and recognition by the cells of a larger number of bacterial antigens, including lipoteichoic acid and peptidoglycan^41^, which consequently triggers an early inflammatory reaction, particularly in response to the highest concentration of PLNC8 αβ (25 µM). Low concentrations of PLNC8 αβ (≤ 6.25 µM) can efficiently inhibit bacterial growth during the first hours of infection, and thus significantly suppress inflammatory effects of the bacteria. These low concentrations did however show a slight (although not significant) increase in cytotoxicity after 24 h. We believe that this increase might be due to the initial suppression of the bacteria, which would allow the keratinocytes to proliferate and increase in numbers. This means that elevated amounts of LDH may be released and registered in the cytotoxicity assay upon new infection of cells. Thus, it is obvious that a final PLNC8 αβ concentration of ≥ 12.5 µM is required for an effective and persistent elimination of the bacteria and thereby prevent their cytotoxicity on host cells. Indeed, a single dose of 12.5 µM of PLNC8 αβ suppressed the growth of bacteria and their cytotoxic effects for 35 h, and a final concentration of 25 µM completely eliminated the bacteria and promoted cell viability > 5 days.

We have recently shown that PLNC8 αβ, in combination with antibiotics, eliminates *Staphylococcus* spp. in a synergistic/additive manner^26^, and in this study these effects were further investigated in a cell culture model. PLNC8 αβ was shown to act, in a dose-dependent manner, additively or synergistically with gentamicin in inhibiting *S. aureus* and its cytotoxic effects on human keratinocytes. Several studies have demonstrated that bacteriocins and antibiotics can synergize to eliminate pathogenic bacteria. For example, Lactoferrin was found to possess antibacterial activity against both susceptible and resistant strains of *S. aureus*, which was improved when combined with different antibiotics^42^. Additionally, Nisin demonstrated synergistic activity with erythromycin and cefazolin against a Group B Streptococcus and mastitis pathogens^43,44^ and we have previously reported that plantaricins EF and JK enhances the effects of various antibiotics against *S. epidermidis*^19^.

The role of NFκB and MAPK (JNK, p38 and ERK) in different human cells processes is well documented^45,46^, including the regulation of apoptosis, proliferation, differentiation and inflammation. Gene expression analysis showed an induction of *c-Jun* and *c-Fos*, indicating a role for MAPK signaling in response to *S. aureus* and PLNC8 αβ. Although gene expression of the NFκB subunits *p50* and *p65* was not affected by the bacteria and the peptides, its involvement in inflammation could not be excluded, since *p50* and *p65* expression does not necessarily reflect NFκB activity, which promted us to further investigate the role of NFκB by using inhibitors. Inhibition of the activity of NFκB, ERK, and JNK, clearly demonstrated the essential role of NFκB in IL-6 and CXCL8 regulation. Blockage of JNK suppressed the accumulation of these inflammatory mediators, and interestingly inhibition of ERK markedly upregulated IL-6 levels. NFκB has previously been shown to be the main regulator of IL-6 expression in T-cells, while regulation of CXCL8 expression is more complex and involves both NFκB and c-Jun via JNK^47^. Parola and colleagues^48^ showed that LPS was more potent than OM-85 (soluble fraction of 21 inactivated bacterial strains) in activating NFκB and inducing IL-6 expression in dendritic cells, whereas CXCL8 expression was equally induced by both LPS and OM-85. Engagement of a specific PRR in the recognition of bacterial antigens is a determining factor in the activation of appropriate intracellular signaling and induction of relevant inflammatory responses. The precise mechanism underlying PLNC8 αβ-dependent modulation of PRRs, signalling molecules and inflammatory responses requires more investigation and is currently ongoing.

In conclusion, we have shown that PLNC8 αβ is effective in improving the survival of keratinocytes when infected with *S. aureus*, by disrupting the bacterial membranes as well as modulating the keratinocytes’ early inflammatory response, and PLNC8 αβ did not negatively affect keratinocyte viability. Our findings suggest that a final concentration of PLNC8 αβ of 3.1 µM is optimal at reducing early cell responses of *S. aureus*-mediated cytotoxicity and inflammation. However, for a complete and persistent elimination of *S. aureus*, a dose of > 12.5 µM is required. This poses the question of whether the administration of multiple doses of a smaller concentration would be preferable to a larger, single-dose administration. Our results also show that PLNC8 αβ in combination with the antibiotic gentamicin acts additively or synergistically, reducing the required dosages of both the peptide and the antibiotic. Thus, we have shown that PLNC8 αβ have the potential to significantly reduce the dose of (and thereby the use of) traditional antibiotics in the treatment of *S. aureus* infections, which would be beneficial in combating the increasing prevalence of antibiotics-resistant bacteria. What would be the optimal dosage and treatment regimen needs to be further examined. As is the case with numerous antimicrobial peptides currently undergoing research, most available research has been performed in in vitro systems. Several studies on AMPs reveal a contextual problem—namely, AMPs not behaving the same in vivo as in vitro. In addition to showing a different behaviour with changing pH and ion concentrations, some AMPs can bind to plasma proteins in serum, which renders the AMPs ineffective^49^. We thus propose further investigation of PLNC8 αβ, where in vivo experiments are a priority. Nevertheless, PLNC8 αβ must be considered as an interesting potential topical treatment for patients with chronically infected wounds.

## Data Availability

The datasets generated and/or analyzed during the current study are available from the corresponding author upon request.
